# Application of a virtual reality-based cognitive-behavioural therapy of social phobia in the treatment of inpatients diagnosed with schizophrenia and social anxiety – a feasibility study

**DOI:** 10.3389/fpsyt.2024.1379541

**Published:** 2024-11-26

**Authors:** Izabela Stefaniak, Adrianna Aleksandrowicz

**Affiliations:** ^1^ Faculty of Medicine, Lazarski University, Warsaw, Poland; ^2^ Experimental Psychopathology Lab, Institute of Psychology, Polish Academy of Sciences, Warsaw, Poland

**Keywords:** social anxiety, schizophrenia, CBT, VRET, Clark and Wells’ cognitive model of social phobia

## Abstract

The co-occurrence of social anxiety symptoms and schizophrenia is a significant clinical problem. So far, social anxiety symptoms have been treated as an integral symptom of schizophrenia, receiving little attention as a target of direct therapeutic interventions. However, some evidence indicates that a high level of social anxiety in this group of patients may be a barrier to the recovery process. This feasibility study evaluated the use of a standard protocol for cognitive-behavioural therapy in the treatment of social phobia, in which social exposure was implemented with the usage of virtual reality (CBT + VRET). The study was conducted in a group of inpatients in a clinical psychiatric unit. Twenty inpatients diagnosed with schizophrenia and comorbid social anxiety symptoms were examined. Eleven patients were assigned to 10 weekly individual CBT+VRET interventions and nine to the control condition. Baseline and post-treatment assessments of social anxiety, psychotic symptoms, stigma, self-esteem, and depressive symptoms were measured before and after intervention. There was a decrease in social anxiety in the CBT+VRET group, while no such decrease was noted in the control group. This study provides preliminary evidence that CBT+VRET is acceptable, safe, and initial data that bears on the question of effectiveness for treating social anxiety disorder in people with schizophrenia. Future research should investigate the efficacy of CBT+VRET for the treatment of social anxiety symptoms and co-morbid schizophrenia in a larger randomised controlled trial.

## 1 Introduction

Over the past few years, the nature of the description and understanding of social anxiety has changed. In DSM-IV, the term *social phobia* was replaced with the term *social anxiety disorder* to reflect the broad and generalised nature of the anxiety (1). According to the DSM-V, the anxiety does not have to be excessive and unreasonable, but it should be considered disproportionate to the actual threat posed by the social situation (2). ICD-10 still highlights the “excessive and unreasonable” elements of the diagnosis. Both diagnostic classifications acknowledge the possible comorbidity with other mental disorders recognizing the complex interplay between these conditions. Categorical classification of mental disorders is associated with various limitations including large within-category heterogeneity, comorbidity, and difficulties in representing subthreshold symptomatology (3).

The presence of social anxiety in psychosis is a phenomenon that is intensively studied and very complex. According to some researchers, both are on a continuum (dimensional context of diagnosis), while others believe they are separate co-occurring disorders (categorical context of diagnosis) (4–6).

Social anxiety may emerge as a part of prodromal symptoms of psychosis (often including social isolation and withdrawal) that could appear long before the onset of psychotic symptoms.

Thirdly, it could be a reaction to the psychosis in terms of a loss of social contacts or a coping strategy in response to perceived threats or other symptoms such as positive, negative, or depressive symptoms (7).

The symptoms of social anxiety and schizophrenia pose numerous clinical challenges. Meta- analyses indicate a frequent co-occurrence of social anxiety symptoms in people with diagnosed schizophrenia with prevalence rates ranging from 8% to 36%, depending on the study (8, 9). Symptoms of social anxiety appear in the period preceding a psychotic episode (10) or in the period after the acute episode of psychosis has subsided (11).

Patients with diagnosed schizophrenia have significantly reduced social activity and often stay isolated (12, 13). The most common reasons for limited social interactions include the presence of negative symptoms, cognitive deficits, persistent and sustained positive symptoms, social anxiety, or social phenomena related to stigma and self- stigma (14).

It has been confirmed that social anxiety symptoms are highly prevalent among outpatients with schizophrenia and adversely affect the duration of untreated psychosis (15). A diagnosis of schizophrenia with comorbid high levels of social anxiety have been connected to an increased risk of suicide (7). Studies also indicate that patients diagnosed with psychotic disorders with comorbid social anxiety symptoms experience higher depression symptoms, lower functioning, lower self-esteem, higher symptom severity, and poorer quality of life (QoL) (9, 16, 17).

The co-occurrence of social anxiety symptoms in patients with experience of psychosis is a complex phenomenon that may involve additional factors that are often absent or less pronounced in patients diagnosed solely with anxiety disorders (18, 19).

One theory attempting to explain the onset of social anxiety symptoms following a psychotic episode is the social status theory (20). This theory states that social anxiety symptoms in patients with psychosis develops in relation to the anticipated loss of social status that follows the experience of psychosis and diagnosis. Perceived stigma makes patients feel ashamed about their diagnosis. Patients become sensitive to how they are socially perceived. They redirect attention to themselves and engage less in social relationships, making this mechanism similar to the cognitive processes that occur in social phobia (21).

New research indicates existing differences between the model of social anxiety in psychosis and the model of social phobia (22). The model of social anxiety in psychosis emphasises cognitive, metacognitive, and behavioural sustaining factors. Higher levels of perceived stigma and shame, lower levels of self-esteem and social rank, and more negative self-appraisals have been highlighted as important cognitive factors. Metacognitive factors include Theory of Mind (ToM), metacognitive mastery, mentalisation, and reasoning biases. The model also takes into account behavioural factors such as defensive behaviours. A model of social anxiety in psychosis has been developed, which leads to a significant sense of threat in social relationships (22).

The literature is lacking in descriptions of therapeutic approaches dedicated to patients with a diagnosis of schizophrenia and co-occurring social anxiety symptoms. Usually, the treatment of social anxiety symptoms is carried out as a part of the treatment of schizophrenia. So far, only a few studies reported a positive effect of group therapy based on cognitive- behavioural therapy (CBT) in this patient group (23–25) or therapeutic interventions carried out online in people with social anxiety symptoms after a first psychotic episode (9).

The standard of treatment for patients with social anxiety, in addition to psychopharmacological treatment (*selective serotonin reuptake inhibitor, serotonin norepinephrine reuptake inhibitor)* is cognitive behavioural therapy (26). Recently, there has been a growing number of studies showing the effectiveness of the combined use of CBT protocols and virtual reality exposure (27, 28). In treating social phobia, it is important to note that virtual reality interventions have been utilized effectively in the treatment of various types of phobias (29).

Virtual reality has also been utilised in studies investigating the treatment of patients diagnosed with schizophrenia. Moreover, previous evidence indicates its usefulness in conducting experimental studies (30) or therapy (31–34). A small number of studies indicate the feasibility of using a standard CBT protocol for social anxiety in patients diagnosed with schizophrenia (23).

This study aimed to evaluate the feasibility (safety and acceptability) of the CBT protocol for social anxiety using virtual exposure to social situations (VRET) to a group of in-patients diagnosed with schizophrenia and coexisting social anxiety symptoms. Moreover, we aimed to report preliminary results of the clinical evaluation of the use of this therapeutic approach in the described group of patients. The severity of social anxiety was assessed before and immediately after therapy in the active and control groups, with other assessment items relating to self-esteem, severity of depression and level of self-stigma.

## 2 Methods

### 2.1 Participants

Participants were inpatients from the Institute of Psychiatry and Neurology in Warsaw (Poland). Patient qualification for therapeutic intervention (individual CBT therapy with VRET) took place among in-patients meeting diagnostic criteria for schizophrenia and social phobia (according to the International Statistical Classification of Diseases and Related Health Problems - ICD-10). Patients were initially diagnosed by an experienced clinician (psychiatrist) and the diagnosis was then confirmed with the Mini International Neuropsychiatric Interview (M.I.N.I PLUS; 35) interview questionnaire. The severity and profile of psychotic symptoms were assessed using the Positive and Negative Syndrome Scale (PANSS; 36), and the severity of social anxiety symptoms was assessed using the Liebowitz Social Anxiety Scale (LSAS). The administration of the detailed assessment for the diagnosis of schizophrenia and social anxiety by an experienced clinician was supposed to eliminate diagnostic errors.

The inclusion criteria were: (1) age: 18–65 years, (2) diagnosis of schizophrenia (ICD-10), (3) concurrent identification of co-occurring traits of social anxiety confirmed by the M.I.N.I PLUS scale and the Leibowitz Scale (>30 points), (4) the patient must give informed consent to participate in the study, (5) stable pharmacological treatment (no modification one month before and during the study).

The exclusion criteria were: (1) addiction to medication and other psychoactive substances (except for nicotine), (2) diagnosis of epilepsy, (3) ophthalmic problems preventing the use of VR, (4) significant mental impairment preventing cooperation during therapy.

The inclusion and exclusion criteria were verified using information obtained from hospital medical records as well as during the interview with the psychiatrist.

### 2.2 Procedures

The study received approval from the Bioethics Committee at the Institute of Psychiatry and Neurology (No 23/2020).

Hospital staff approached all potential participants. Those who agreed to participate and met the inclusion and exclusion criteria were recruited and assigned to either the control or experimental group. Patients were given information about the study and signed an informed consent form before entering the study. At baseline, participants were assessed with clinical interviews by an experienced psychiatrist. The outcome measures were assessed at pre-treatment and post-treatment. Then, individuals were allocated into one of the two study groups.

### 2.3 Treatment conditions

The control group received the interventions offered in the unit. The programs were delivered by qualified psychologists or psychiatrists. These are standard programs delivered in the unit. Details on the program are listed in **Table 1**. The active group received the interventions offered in the unit as well as individual CBT for social phobia with VRET. Additionally, both groups received pharmacological treatment.

**Table 1:** Therapeutic interventions present in the therapeutic program of the unit.

Description	Information on the type of therapeutic interventions in the unit
Duration of stay 70 days	Community meeting
	Physical activity classes
	Group psychotherapy
	Individual psychotherapy
	Initiative workshops
	Metacognitive training
	Psychoeducation

The active group received individual CBT with VRET. In the standard treatment protocol for social phobia, social exposures are planned in two forms: in imagination and in real life (26). Exposure to virtual reality is a good solution for hospitalised patients when they are unable to leave the ward. Virtual exposure provides access to a variety of social situation scenarios. Studies show that exposure to virtual social environments reduces levels of social anxiety (37, 38). In the protocol used in the study described herein, social exposures of real life/imagination were replaced with exposures in virtual reality. The scenarios address the following social situations:

- Job interview
- Social conversation
- Public speaking in the lecture hall
- Speaking at a meeting in the conference room
- Buying a ticket at a railway ticket office
- Train compartment
- Visit to a restaurant
- Return of goods to a shop
- Phone call in a public place

Each proposed virtual reality social scenario can be recreated at three difficulty levels (from easy through medium to difficult). In the easy scenario, the patient observes the virtual social environment. At the “medium” level of the scenario, the patient’s task is to interact in the virtual world, while at the “difficult” level the patient is exposed to having to deal with some kind of interaction difficulty. The VR Mind system using the HTC VIVE virtual helmet was used for the study. The VR Mind software consisted of nine therapy scenarios. **Table 2** presents details of each therapy session of the active condition.

**Table 2:** Program of individual therapy for social phobia offered to patients in the active group.

Session (55 min.)	Description of the session
1	1. Greeting. 2. Presentation of general information about the therapy. 3. Identification of problems and therapy goals. 4. Discussion of patient expectations. 5. Summarising the session and signing the therapeutic contract.
2	1. Greeting. 2. Introducing the patient to Wells’ cognitive model of anxiety. 3. Psychoeducation on social anxiety. 4. Assignment of personal task. 5. Summary of the therapy
3	1. Greeting. 2. Discussion of the personal task. 3. Introducing the cognitive model of social anxiety. 4. Assignment of personal task. 5. Summary.
4	1. Greeting. 2. Discussion of the personal task. 3. Psychoeducation on cognitive distortions. 4. Introducing cognitive restructuring – discussing negative automatic thoughts/conceptions. 5. Personal task. 6. Summary.
5	1. Greeting. 2. Discussion of the personal task. 3. Introduction to behavioural techniques. 4. First VR exposure – the therapist chooses one of the exposures from the basic or medium level, e.g. a visit to a restaurant. 5. Personal task. 6. Summary.
6–9	1. Greeting. 2. Discussion of the personal task. 3. Exposure in virtual reality (VR). 4. Discussion of the virtual reality exposure. 5. Personal task. 6. Summary.
10	1. Greeting. 2. Discussion of the personal task. 3. Summary of the therapy. 4. End of therapy.

### 2.4 Assessment tools

#### 2.4.1 Clinical assessment

PANSS (Positive and Negative Syndrome Scale) (36) – a scale used to measure symptom severity in patients with schizophrenia. Symptoms are grouped into subscales: positive symptoms, negative symptoms and scales of general psychopathology. The assessment is made on a scale of 1 (no symptom) to 7 (extremely severe symptom severity) for 30 different items.

#### 2.4.2 Questionnaires

1. Liebowitz Social Anxiety Scale (LSAS) (39) – the questionnaire includes two subscales – the subscale of anxiety and the subscale of avoidance. Each subscale consists of 12 items. Each of these has responses recorded on a 4-point Likert scale. The anxiety subscale ranges from 0 (no fear) to 3 (strong fear). The avoidance subscale has the same range and is based on the percentage of times the patient avoids a specific situation (0 – never; 1 – sometimes; 2 – often; 3 – always). The scale can be used to screen for the prevalence of social anxiety in a group of people with diagnosed schizophrenia.
2. The Internalized Stigma of Mental Illness (ISMI) (40) – the ISMI scale contains 29 items to calculate scores on five subscales and a total score. Each item on the scale is accompanied by answers on a 4-point Likert scale. The specific subscales are alienation (6 items), stereotype endorsement (7 items), perceived discrimination (5 items), social withdrawal (6 items), and stigma resistance (5 items).
3. Rosenberg Self-esteem Scale (RSES) (41) – a 10-item scale that measures global self-esteem by measuring both positive and negative feelings about oneself. All questions are answered using a 4-point Likert scale format from strongly agree to strongly disagree.
4. Social and Occupational Functioning Assessment Scale (SOFAS) – this is a scale developed as a tool to assess Axis V according to DSM-IV. SOFAS is a global rating of current functioning ranging from 0 to 100, with lower scores representing poorer functioning.
5. Calgary Depression Scale for Schizophrenia (CDSS) (42) – it is widely used to assess the severity of depression in schizophrenia. In addition, depressive symptoms as assessed on the CDSS were found to be strongly correlated with patient-rated severity of illness in schizophrenia (43). The scale has 9 items rated using a 4-point scale (0 – negative, 1 – mild, 2 – moderate, 3 – severe).

### 2.5 Main assessment points

The study proposed two study points: T0 (before patients were allocated to groups) and a T1 point (immediately after the therapeutic intervention). Assessment points are presented in **Table 3**.

**Table 3:** Table showing the assessment points of the subjects.

Assessment point **(T0)**	Overall assessment of the patient by the psychiatrist • M.I.N.I PLUS (*MINI-International Neuropsychiatric Interview, Polish Version*) • PANSS (*Positive and Negative Syndrome Scale*) • LSAS (*Liebowitz Social Anxiety Scale*) • ISMI (*Internalized Stigma of Mental Illness*) • RSES (*Rosenberg Self-esteem Scale*) • SOFAS (*Social and Occupational Functioning Assessment Scale*) • CDSS (*Calgary Depression Scale for Schizophrenia*)	
Sessions from 1 to 10	Active group CBT +VRET therapy and participation in therapy offered in the unit.	Control group Without social phobia therapy, participation in the therapy offered at the unit
**(T1)**	Reassessment of the patient made after therapeutic intervention: • PANSS (*Positive and Negative Syndrome Scale)* • LSAS (*Liebowitz Social Anxiety Scale*) • ISMI (*Internalized Stigma of Mental Illness*) • RSES (*Rosenberg Self-esteem Scale*) • SOFAS (*Social and Occupational Functioning Assessment Scale)* • CDSS (*Calgary Depression Scale for Schizophrenia*)	

### 2.5 Analysis

Data expressed on a quantitative scale were presented as mean ± SD. Differences between the post-therapy point and the pre-therapy point were calculated. Data expressed on a qualitative scale were presented as the number and percentage of the sample. The effect of therapy was determined by assessing acceptability (defined as the percentage of offered sessions that were attended) and safety (defined as the number of severe adverse events related to the treatment, including inpatient admissions, suicide attempts, or other life-threatening events).

## 3 Results

### 3.1 Course of the study

### 3.2 Study group

**Figure 1:**
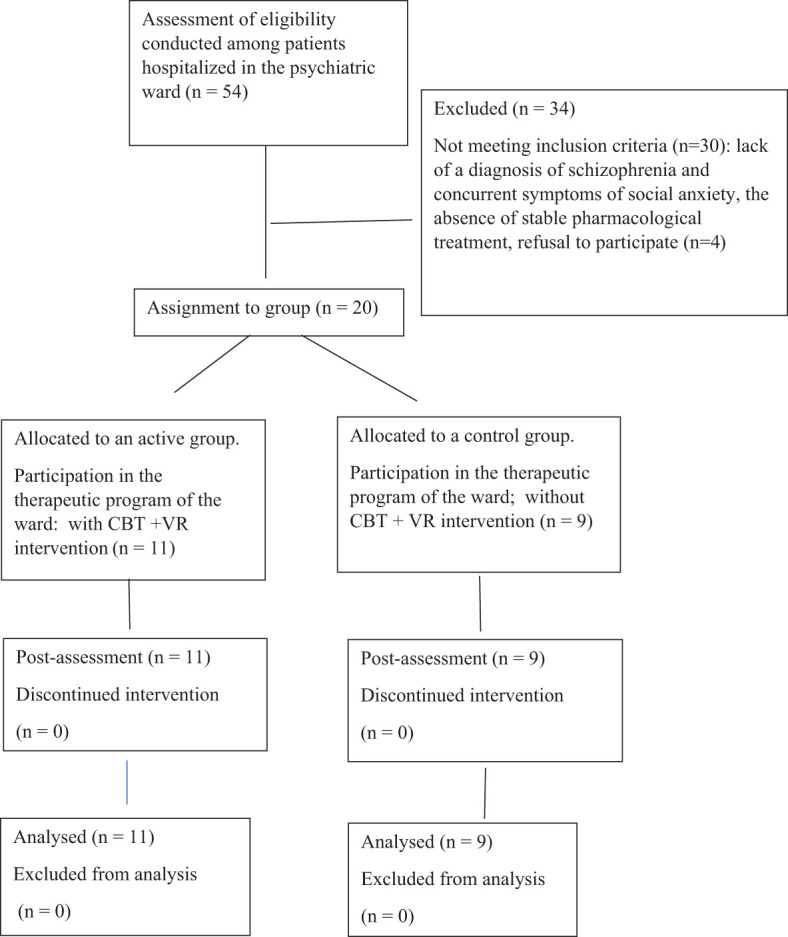
Diagram showing the course of the study.

Recruitment took place from 2020 to 2022. Of 54 patients recruited, 11 were allocated to the active group and 9 to the control group (**Figure 1**). The mean age of the subjects was 37 years (SD=7.17), and the mean duration of the disease was 11.3 years (SD=6.78). Men comprised 75% of the subjects (n=15) and women 25% (n=5). The majority of participants were on a pension (n = 12), n=4 were employed and n=2 were unemployed. The participants’ marital status included 16 single individuals, 2 who were married, and 2 who were divorced. One patient had vocational education (n = 1), n=8 secondary education, and n=1 higher education (n = 11). All subjects in the active and control groups received pharmacological treatment according to previous medical recommendations, which were not modified one month before starting therapy and during the therapy proposed in the study.

All individuals met the criteria for schizophrenia and social phobia according to the ICD-10.

### 3.3 Acceptability (defined as the percentage of offered sessions that were attended)

The participants attended all the scheduled sessions. Two participants had their VR exposure replaced with exposure in imagination due to a VR software failure. We observed that patients were eager to engage in the therapy and willingly attended subsequent sessions without terminating the therapy prematurely. It is important to note that the therapy was an additional activity for the patients and was conducted after completing other therapeutic interventions.

Cognitive-behavioral therapy with VR exposure in the treatment of social anxiety symptoms appears to be a highly acceptable method for treating individuals diagnosed with schizophrenia.

### 3.4 Safety (defined as the number of severe adverse events related to the treatment, including inpatient admissions, suicide attempts, or other life-threatening events)

No severe adverse events were reported during the treatment. Patients freely chose the exposure scene and there were no instances of premature removal of the goggles (the duration of exposure was approximately 10 minutes). Specifically, there were no suicide attempts or psychosis crises.

### 3.5 Modifications of CBT for social anxiety disorder for people with schizophrenia.

Overall, the treatment did not differ from the standard protocol, including individualised conceptualization, safety behaviours experiment, core belief work, and creating a blueprint. All participants received the core components of CBT for social anxiety disorder.

As is standard for CBT for social anxiety disorder, the focus was on exposure exercises. Instead of the standard real-life exposure (in the unit), patients underwent exposure to virtual reality.

During therapeutic work, we observed that the greatest differences concerned the content of key beliefs about oneself and others compared to individuals with social anxiety disorder alone.

Patients with schizophrenia more frequently held beliefs in which they saw themselves through the lens of their diagnosis (self-stigmatisation: “I’m worthless because I’m sick”, “I am sick and it shows”), or beliefs about others being threatening (influence of persecutory attitudes). It had implications for psychoeducation and cognitive modification. A common element, however, was exposure and the habituation mechanism to triggering social factors.

### 3.6 The liebowitz social anxiety scale - outcomes

The primary measurement point was the changes in the Liebowitz Social Anxiety Scale (LSAS) between T0 (pre-) and T1 (posttreatment), in the active and control groups. Below are the tables with the results obtained on the Liebowitz Social Anxiety Scale: anxiety subscale (LSAS- A) (**Table 4**), avoidance subscale (LSAS-Av) (**Table 5**), and total scale (LSAS) (**Table 6**).

**Table 4:** Scores of the social anxiety subscale of the LSAS scale.

LSAS (A)					
	Group	M	SD	Difference	Std. dev. Difference
*LSAS (A) T0*	*Active*	*47.273*	*13.943*		
*LSAS (A)T1*	*Active*	*40.454*	*18.806*	*6.818*	*8.1831*
LSAS (A) T0	Control	33.400	24.214		
LSAS (A) T1	Control	28.600	30.427	4.800	13.809

M, mean; SD, standard deviation; LSAS, Liebowitz Anxiety Scale; LSAS (A), anxiety subscale of the LSAS scale.

**Table 5:** Scores of the social avoidance subscale of the LSAS scale.

LSAS (Av)					
	Group	M	SD	Difference	Std. dev. Difference
*LSAS (Av) T0*	*Active*	*45.364*	*10.308*		
*LSAS (Av) T1*	*Active*	*34.727*	*15.869*	*10.636*	*10.773*
LSAS (Av) T0	Control	33.00	20.187		
LSAS (Av) T1	Control	31.400	22.131	1.6000	5.272

M, mean; SD, standard deviation; LSAS, Liebowitz Anxiety Scale; LSAS (Av), avoidance subscale of the LSAS scale.

**Table 6:** Total social anxiety score of the LSAS scale.

LSAS (TOT)					
	Group	M	SD	Difference	Std. dev. Difference
*LSAS (TOT) T0*	*Active*	*92.636*	*22.848*		
*LSAS (TOT) T1*	*Active*	*75.182*	*33.295*	*17.454*	*17.392*
LSAS (TOT) T0	Control	66.400	43.912		
LSAS (TOT) T1	Control	60.000	52.019	6.4000	19.060

M, mean; SD, standard deviation; LSAS, Liebowitz Anxiety Scale; LSAS (TOT), total LSAS scale score.

### 3.7 The secondary outcomes

The secondary outcomes were the changes in the PANSS (Positive and Negative Syndrom Scale), ISMI (The Internalized Stigma of Mental Illness), RSES (Rosenberg Self-esteem Scale), SOFAS (Social and Occupational Functioning Assessment Scale), CDSS (The Calgary Depression Scale for Schizophrenia) between T0 and T1, in the active and control groups. Below are the tables with the results (**Table 7**).

**Table 7:** Results of the secondary outcomes.

Scale	Group	M	SD	Difference	Std. dev. Difference
PANSS	Active T0	46.625	8.8307		
	Active T1	39.875	5.5918	6.7500	12.279
	Control T0	71.714	13.949		
	Control T1	61.428	14.547	10.286	1.0610
ISMI	Active T0	2.4890	0.3645		
	Active T1	2.2539	0.3085	0.2351	0.3767
	Control T0	2.0985	0.4641		
	Control T1	1.9360	0.5583	0.1626	0.4503
RSES	Active T0	22.00	3.8210		
	Active T1	20.636	4.2491	1.3636	3.5573
	Control T0	17.857	4.0591		
	Control T1	15.714	3.3523	2.1428	3.6710
SOFAS	Active T0	68.818	13.385		
	Active T1	73.909	11.022	10.784	
	Control T0	37.286	19.015		
	Control T1	56.143	10.946	25.472	
CDSS	Active T0	8.7	4.6916		
	Active T1	6.0	4.1366	2.7	5.5186
	Control T0	8.0	4.8990		
	Control T1	6.0	4.4272	2.0	3.7947

M, mean; SD, standard deviation; PANSS, Positive and Negative Syndrom Scale; ISMI, The Internalized Stigma of Mental Illness; RSES, Rosenberg Self-esteem Scale; SOFAS, Social and Occupational Functioning Assessment Scale; CDSS, The Calgary Depression Scale for Schizophrenia.

## 4 Discussion

The study investigates the initial feasibility of a cognitive behavioural therapy protocol for co- morbid social anxiety symptoms in in-patients diagnosed with schizophrenia and social anxiety. Our aim was to investigate acceptability, safety and to compare preliminary changes in psychopathological symptoms between pre- and post-intervention. The co-occurrence of both diagnoses is widespread and represents a significant clinical problem and therapeutic challenge (44). Until recently, symptoms of social anxiety occurring in people with schizophrenia were treated as an integral part of the diagnosis of schizophrenia. The application of therapy aimed at treating social anxiety symptoms in a group of psychotic patients is a novel approach.

Recently, there have been few studies indicating the applicability of a cognitive behavioural protocol for social anxiety symptoms in a group of people with psychosis e.g., a case study (45) and research on a larger group of patients (23, 24). A meta-analysis by Michail et al. (46) and a meta-analysis by Heavens et al. (47) described studies on the application of CBT in the treatment of anxiety in patients with psychosis. The meta-analyses indicated that studies on the use of CBT in treating anxiety in patients with psychosis showed beneficial effects. Both reviews underscored a need for scientifically rigorous studies in this area. Further research is required, including well-designed randomised controlled trials (RCTs).

The current feasibility trial aimed to evaluate the utility of implementing CBT with VRET on a sample of patients diagnosed with schizophrenia and to confirm that the randomised controlled trial could be a potential direction for future studies. The recruitment and adherence rates confirm that inpatients diagnosed with schizophrenia show interest in enrolling in a clinical trial. Thus, the current trial offers a promising path for future research.

The main objective of the current study was to conduct a CBT+VR intervention among patients hospitalised in a psychiatric ward. This study design proved to be very challenging due to the limited number of patients meeting the inclusion criteria. Patients recruited for the study were those staying in the psychiatric ward. Their hospitalisation period was three months. In practice, this meant individual patients meeting the inclusion criteria for the study. An additional challenge was meeting the criterion for stable pharmacological treatment. We observed that most hospitalised patients had their pharmacological treatment modified. The described difficulties resulted in only a few individuals meeting the inclusion criteria during a single stay, making it impossible to conduct a randomised study on a larger sample of patients. The study location and the complex inclusion criteria proved significant obstacles in conducting the study.

The proposed CBT+VR protocol was an attractive form of therapy for hospitalised patients diagnosed with schizophrenia. All patients who participated in the study emphasised the interesting aspect related to the use of VR. Patients indicated that this type of therapy was a new experience for them compared to prior treatments. Virtual reality exposure was an excellent solution for hospitalised individuals who had difficulty finding diverse situations for social exposure. Patients were eager to participate in the exposure sessions. All patients qualified for the active group completed the therapy process. The results of our research on the acceptability of the CBT+VR protocol confirm the findings from previous studies (32, 48). Similarly many studies utilising virtual exposure recognized the benefits of using this tool in the hospitalised patients (49). Tapping into virtual reality for exposure therapy offers numerous advantages. Incorporating VR into therapy can enhance the ease, acceptability, and safety of anxiety treatment. Virtual reality exposure therapy allows for individualised, gradual, controlled, immersive exposure, which is easy to implement for therapists and often more acceptable to patients than *in vivo* or imaginal exposure (50).

One major challenge concerning the transportability of interventions to other settings is the lengthy three-month duration of inpatient stays, which may restrict the feasibility of relying solely on inpatient interventions. Consequently, future research should address the unique challenges associated with various conditions in psychiatric hospitals and the implications of length of stay. It would be beneficial to explore the development of more concise inpatient programs or integrated approaches that combine inpatient care with subsequent outpatient sessions.

The severity of social anxiety was measured between the pre-intervention point and the post- intervention point was analysed in the experimental and control groups. Our study indicates visible changes in the avoidance subscale and the total LSAS scale. These studies indicate promising results in the form of reduced severity of social anxiety symptoms. Results concerning the severity of symptoms of social anxiety are consistent with data from other studies (23, 24). Changes in other scales used in the study (PANSS, ISMI, RSES, SOFAS, CDSS) showed no visible changes. However, the current study presents only preliminary descriptive results that should be further verified.

Patients hospitalised in our ward had experienced a psychotic crisis. Despite improvement in psychotic symptoms, many of them continued to struggle with functioning difficulties. Social anxiety symptoms are one of the factors inhibiting the recovery process (51, 52). The proposed intervention could be one way to address this problem.

Social anxiety in people diagnosed with schizophrenia has a more complex nature than among patients without a diagnosis of schizophrenia (22). Working with this group of patients requires considering beliefs related to self-stigmatisation and delusional beliefs, which presents a significant clinical challenge.

The proposed study showed that patients accepted both the CBT protocol for social anxiety therapy and the virtual exposures conducted as part of the therapy. We did not observe any drop-outs in our group. The method was safe for hospitalised patients diagnosed with schizophrenia. Due to the use of virtual reality, maintaining constant and ongoing contact with technical support personnel is crucial. The lack of such collaboration results in the need to interrupt therapy conducted according to the protocol and switch from virtual exposure to either real-life exposure or imaginal exposure. Such situations also constituted a reason for excluding patients from our study.

This study, similarly to previous research, demonstrates the feasibility, acceptability and tolerability of using a cognitive behavioural therapy protocol targeting social anxiety symptoms supported by VRET in people with diagnosed schizophrenia and social phobia (23, 53).

## 5 Limitations of the study

The current study has its limitations. First of all, this study was conducted on a small group of people. This is related to the limited access to patients with both diagnoses who were hospitalised at the time. A sample (n=20) of 54 approaches was examined, reflecting the challenges of meeting the inclusion and exclusion criteria for the study.

A major problem for the study presented was assembling the study group, which significantly affected its implementation. The conditions under which the study was conducted hindered the implementation of a randomised clinical trial. It should be noted that despite the initial goal of conducting a clinical trial, the current study was not structured to yield efficacy results. It was not a randomized clinical trial, and the baseline scores in the two treatment groups highlight the limitations in comparing these groups. Additionally, the limitation could be not implementing qualitative interviewing as it could provide further information about the results of this pilot trial.

Another limitation is the access and operation of the system, which enables virtual reality exposure.

In the current study, we implemented a range of inclusion and exclusion criteria to ensure the reliability of the tested sample of patients and avoid potential confounding variables.

However, due to the relatively small sample sizes per group, the statistical analyses are unreliable and studies on larger samples are needed to perform statistical tests. The current feasibility trial aimed to evaluate the utility of implementing CBT with VRET on a sample of patients diagnosed with schizophrenia and to confirm that the randomized controlled trial could be a potential direction for future studies. Moreover, the recruitment and adherence rates confirm that inpatients diagnosed with schizophrenia show interest in enrolling in a clinical trial.

## 6 Conclusions

The results of the current study provide a promising path for future research. The conclusion of the study suggests that it is worth looking at the possibility of using an established and well-proven therapeutic protocol to treat people with social anxiety symptoms in schizophrenia. However, this requires attention to the possibility of co-occurrence of both disorders, a look at the etiology of social phobia in this group of patients, and above all, a targeted and purposeful psychotherapeutic treatment of each condition. A randomised clinical trial conducted on a sufficiently large group of patients is needed to conclude the efficacy of such therapy. Future research should replicate the current design by implementing a longitudinal design with follow-up measurements over a more extended period Moreover, the intention-to-treat (ITT) analysis would strengthen the interpretation of the results. The conclusions from our study support the social phobia model in individuals diagnosed with psychosis described by (22). Future research should aim to develop a modified theoretical model of social anxiety in individuals with psychosis, which would serve as a basis for therapeutic work.

## Data Availability

The raw data supporting the conclusions of this article will be made available by the authors, without undue reservation.
